# Study approach and field work procedures of the MentDis_ICF65+ project on the prevalence of mental disorders in the older adult European population

**DOI:** 10.1186/s12888-017-1534-5

**Published:** 2017-11-16

**Authors:** Jana Volkert, Martin Härter, Maria Christina Dehoust, Holger Schulz, Susanne Sehner, Anna Suling, Karl Wegscheider, Berta Ausín, Alessandra Canuto, Mike J. Crawford, Chiara Da Ronch, Luigi Grassi, Yael Hershkovitz, Manuel Muñoz, Alan Quirk, Ora Rotenstein, Ana Belén Santos-Olmo, Arieh Y. Shalev, Jens Strehle, Kerstin Weber, Hans-Ulrich Wittchen, Sylke Andreas

**Affiliations:** 1 Department of Medical Psychology, University Medical Centre Hamburg-Eppendorf, Hamburg, Germany; 2 Department of Psychosocial Prevention, University of Heidelberg, Bergheimer Str. 56, D-69115 Heidelberg, Germany; 3 Institute of Medical Biometry and Epidemiology, University Medical Centre Hamburg-Eppendorf, Hamburg, Germany; 4 School of Psychology, Complutense University of Madrid, Madrid, Spain; 5 Nant Foundation, East Vaud Psychiatric Institute, Route de Nant, Corsier-sur-Vevey, Switzerland; 6 Royal College of Psychiatrists, London, UK; 7 Institute of Psychiatry, Department of Biomedical and Specialty Surgical Sciences, University of Ferrara, Ferrara, Italy; 8 Hadassah University Medical Centre, Jerusalem, Israel; 9 Department of Psychiatry, NY Langone Medical Centre, New York, USA; 10 Institute of Clinical Psychology and Psychotherapy, Technische Universität Dresden, Dresden, Germany; 11 Medical Direction, University Hospitals of Geneva, Geneva, Switzerland; 12 Department of Psychiatry and Psychotherapy, Ludwigs-Maximilians-Universität München, Nußbaumstr. 7, 80336 Munich, Germany; 13 Institute of Psychology, Alpen-Adria University Klagenfurt, Klagenfurt, Austria; 14 Department of Psychology, University Witten/Herdecke, Alfred-Herrhausen-Str. 50, 58448 Witten, Germany

**Keywords:** Mental disorders, Prevalence, Old age, Methodology

## Abstract

**Background:**

This study describes the study approach and field procedures of the MentDis_ICF65+ study, which aims to assess the prevalence of mental disorders in older adults.

**Methods:**

An age-appropriate version of the Composite International Diagnostic Interview (CIDI65+) was developed and tested with regard to its feasibility and psychometric properties in a pre-test and pilot phase. In the cross-sectional survey an age-stratified, random sample of older adults (65–84 years) living in selected catchment areas of five European countries and Israel was recruited.

**Results:**

*N* = 3142 participants (mean age 73.7 years, 50.7% female) took part in face-to-face interviews. The mean response rate was 20% and varied significantly between centres, age and gender groups. Sociodemographic differences between the study centres appeared for the place of birth, number of grandchildren, close significants, retirement and self-rated financial situation. The comparison of the MentDis_ICF65+ sample with the catchment area and country population of the study centres revealed significant differences, although most of these were numerically small.

**Conclusions:**

The study will generate new information on the prevalence of common mental disorders among older adults across Europe using an age-appropriate, standardized diagnostic instrument and a harmonized approach to sampling. Generalizability of the findings and a potentially limited representativeness are discussed.

## Background

In Western countries, the population of individuals older than 65 is predicted to rise from 16% in 2010 to over 26% in 2050 [1]. Aging is associated with increasing frequency of disease and the need for care and service utilization leads to rising costs for healthcare systems [2]. The International Classification of Functioning, Disability and Health (ICF, [3]) is a bio-psycho-social model that offers a comprehensive framework for understanding the health status of older people with mental disorders [4, 5]. The ICF comprises 7 components to comprehensively assess an individual’s health status and the related factors of health, disability and functionality [3]. The component “health condition” is used to describe mental disorders based on the International Classification of Diseases (ICD-10, [3]). The component “body functions and structures” adds information on symptom severity, the course of a disorder and prognostic factors. The two components “activities” and “participation” include quality of life and activities and participation in everyday life and society. “Environmental factors” include, for example, access to and the cost of health care services. The component “personal factors” takes sociodemographic characteristics, such as age, gender and family status, into account.

In addition to the importance of understanding older people’s health status based on a comprehensive model, empirical findings on the epidemiology of mental disorders in old age are also urgently needed. So far, studies that have investigated the prevalence, symptom severity and course of mental disorders in older people are scarce and heterogeneous [6–8]. Most studies report decreased prevalence rates in older people >65 years [9, 10] and those aged >80 years [10], and have focused on dementia and depression [11–13]. The heterogeneity of previous findings may be associated with methodological issues, particularly a lack of feasible and age-appropriate standardized instruments to diagnose mental disorders in older adults [14–16]. Against the background of this lack of knowledge and the heterogeneous findings on common mental disorders in older adults, the MentDis_ICF65+ study aims to 1) adapt diagnostic instruments for older adults, 2) assess the psychometric properties of an adapted and translated standardized/structured diagnostic interview, and 3) collect data on the point, 1-year and lifetime prevalence of mental disorders in the older adult population of different European countries and Israel and assess the relationship with symptom severity, quality of life, level of functioning and service utilization. Because dementia has already been extensively assessed in previous studies and age-appropriate measures exist for this disorder, it is not included in this study. Accordingly, the following research questions (RQ) were derived:

- **RQ 1:** How feasible is an adapted version of a standardized diagnostic interview for the needs of people aged 65 and above in different European countries and Israel?
- **RQ 2:** What are the psychometric properties of the adapted and translated standardized diagnostic interview?
- **RQ 3:** What are the point, year and lifetime prevalence rates of mental and physical disorders among the older adult population of different European countries and Israel, and what is the relationship of this prevalence to symptom severity, activities and participation and service utilization?

This paper presents background information on the pre-test (RQ 1) and pilot test (RQ 2) prior to a detailed description of the cross-sectional study approach and field procedures (RQ 3) of the MentDis_ICF65+ project.

## Method

### Design

**Fig. 1:**
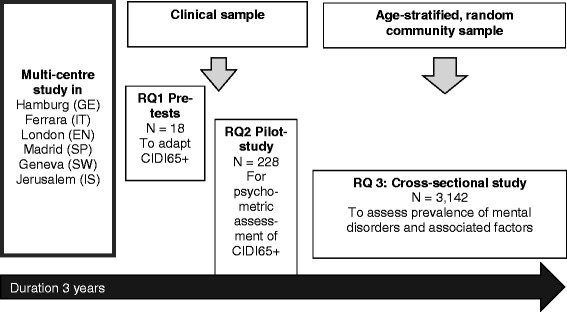
Design of multi-stage and multi-centre MentDis_ICF65+ study. *Detailed legend*: RQ = Research Question; GE = Germany, IT = Italy, EN = England, SP = Spain, SW = Switzerland, IS = Israel; CIDI65+ = Composite International Diagnostic Interview for Adults aged 65 years and above

The MentDis_ICF65+ study has a stepwise cross-sectional design (see Fig. 1 [17]) to address the three research questions listed above:

#### Feasibility of the CIDI65+ (RQ 1)

To answer **RQ1,** a pre-test phase was conducted. This phase was devoted to the adaptation of an age-specific assessment tool (e.g., Composite International Diagnostic Interview, CIDI) and to the translation and back translation of this instrument for older people.

#### Psychometric properties of the CIDI65+ (RQ 2)

To answer **RQ2**, a pilot phase was conducted in all study centres as part of the adaption and psychometric assessment of the diagnostic instrument prior to the cross-sectional study.

#### Main prevalence study (RQ 3)

To answer **RQ3**, a cross-sectional, multi-centre study was conducted based on age- and gender-stratified random samples of individuals aged 65 to 84 years living in communities in selected catchment areas of five European countries — Hamburg (Germany), Ferrara (Italy), London (England), Madrid (Spain), Geneva (Switzerland) — and Jerusalem (Israel).

### Sampling

#### Feasibility of the CIDI65+ (RQ 1)

Older participants from two study centres (London, England and Hamburg, Germany) were recruited to test the feasibility of the adopted instrument. Each site sought to collect a heterogeneous sample that was equally distributed across two age groups (65–74 and 75–84 years), both with and without mental disorders.

#### Psychometric properties of the CIDI65+ (RQ 2)

Each study centre recruited a convenience sample of older in- and outpatients with different mental and physical disorders. The participants were informed about the purpose of the study to assess the quality of the CIDI65+ interview by participating in a test-retest study with ideally a 3-day interval between the 1st and 2nd interview [18].

#### Main prevalence study (RQ 3)

The two-stage sampling approach of the cross-sectional survey to assess prevalence rates of mental disorders in older people was defined a priori. To achieve comparability of samples between the study centres and to allow for a similar power across all age and gender groups, two strata for age and gender were defined. Two equally large age groups were formed for 65 - <75 and 75 - <85 year olds. An analogue for the second stratum gender was created, where half of the cohort was male or female, respectively. The criteria for the definition of the catchment areas were locations in an adjacent area to each study centre and needed to maximize the representativeness for the old-age population of the country. In Switzerland, the sample was also stratified according to socioeconomic status.

The inclusion criteria for the participants included the ability to provide informed consent, living in the predefined catchment area at the beginning of the cross-sectional study, and being 65 to 84 years old. The exclusion criteria included severe cognitive impairment as assessed with the MMSE (Mini-Mental State Examination, cut-off score > 18 [19])^1^ and insufficient ability to communicate in the language in which the interview was conducted. Nursing home residents were included in the sample if their place of residence was officially registered or their postal addresses had been made available to market research companies and they met the inclusion criteria.

The response rate was defined as the total percentage of participants with completed interviews in the study from those who were contacted with an invitation letter [20, 21]. In most study centres (Ferrara, Geneva, Jerusalem, London and Madrid), the written invitation letter was followed by a phone call to ask potential participants if they were willing to take part in the study. Due to the ethics regulations in Hamburg, people had to write back to demonstrate their interest in participating (no phone calls). The study was approved by the research ethics committees in all six participating countries [17].

### Measures

Instruments were selected to cover the domains of the International Classification of Functioning, Disability and Health (ICF) model [3].

#### The Composite International Diagnostic Interview for older adults (CIDI65+)

An age-appropriate, computerized version of the fully structured lay interview Composite International Diagnostic Interview (CIDI [22]), the CIDI65+, was developed by the study group [18] for use in the older adult population to diagnose Axis I mental disorders according to the Diagnostic and Statistical Manual of Mental Disorders (DSM) version IV criteria [23]. The CIDI [22] was adapted to the social, cognitive, and psychological abilities and needs of older adults and evaluated the syndrome domains of Axis I mental disorders [18]. The process of adapting the interview to the unique conditions of older adults comprised several aspects, including adding words, offering alternative questions and detailed section introductions, breaking down long questions into less complicated questions, sensitizing scales upfront and embedding a fuller spectrum of syndromes. The English paper–pencil version was translated into German, Spanish, Hebrew, Italian and French with a back-translation and was then computerized. The questions were administered by trained lay interviewers using a computer-assisted personal interview (CAPI) version of the CIDI65 + .

#### The Health of the Nation Outcome Scales65+ (HoNOS65+)

The Health of the Nation Outcome Scales for older people (HoNOS65+) [24] is a 12-item clinician-assessed instrument to assess the severity level of 12 problem areas of mental health in older people (e.g., item 2: self-harm; item 7: depressive mood). Items are scored on a scale from 0 (no problem) to 4 (severe or very severe problems). The HoNOS65+ has a comprehensive glossary with anchor examples for scoring. The instrument is one of the most commonly used scales for older people who are treated for psychiatric issues, with mostly satisfactory psychometric properties [25, 26].

#### The short version of the Big Five Inventory (BFI-10)

The short version of the Big Five Inventory (BFI-10) — based on the 44-item version of the BFI by Rammstedt and John [27] — was used to assess personality as another important component of the ICF. The BFI-10 covers the five personality domains of extraversion, agreeableness, conscientiousness, neuroticism, and openness. Items are rated on a five-point Likert scale (1 = disagree strongly to 5 = agree strongly). The BFI-10 has satisfactory psychometric properties [27].

#### The World Health Organization QoL assessment (WHOQoL-BREF)

The 26 items of the WHO Quality of Life short version [28] was used to measure quality of life. The WHOQoL-BREF was developed by the World Health Organization [28] from the WHOQoL-100 item version. The self-report questionnaire assesses the individual’s quality of life, including physical and psychological well-being, environmental factors and social support, while also taking into account the context of culture and value systems, personal goals, standards and concerns. The psychometric properties are satisfactory [29], and there is evidence that the WHOQOL-BREF is appropriate for older people [30].

#### World Health Organization Disability Assessment Schedule II (WHODAS II)

To assess activities and participation according to the ICF, the 12-item self-report version of the World Health Organization Disability Assessment Schedule II (WHODAS II, [31]) was used. The WHODAS II measures the functional impairment of daily activities in six different areas (including communication and self-supply). Satisfactory psychometric scores for patients with affective disorders are reported regarding reliability and validity [32], and first evidence that the WHODAS II is an adequate instrument for assessment in old age is available [33].

### Procedure (RQ 3)

Data quality control was implemented locally at each study centre and centrally at the coordinating study centre (Hamburg, Germany) to ensure reliability, validity and timeliness of the data. All completed interviews were transmitted electronically to the coordinating study centre for final checking and storage. Data checks were conducted among individual participants for completion status, identification number, consistency in the questionnaire variables, and length of the interview. Furthermore, data checks were completed across variables, interviewers and study centres.

All interviewers had completed the same standardized 2-day training carried out by WHO-certified trainers and adhered to the same study protocol regarding contacts and interview administration (the interviewer cross-sectional study protocol can be obtained from the authors upon request).

### Statistical analyses

#### Feasibility of the CIDI65+ (RQ 1)

To test the feasibility of the adapted instruments, multiple methods were applied, including a respondent and interviewer debriefing, behaviour analysis and desk-based review. The methods’ performance was evaluated by categorizing and comparing the number, type and severity of problems detected by each method.

#### Psychometric properties of the CIDI65+ (RQ 2)

To assess the psychometric properties of the CIDI65+, we calculated test-retest reliabilities (as agreement in categorical variables) using the kappa statistic [34, 35]. Kappa values of less than .40 were considered poor agreement, values between .40 and .60 were fair, and values between.61 and 0.76 were considered good or excellent agreement. Intraclass correlation coefficients (ICC) were calculated to derive agreement estimates for continuous variables (e.g., age of onset, duration) [18].

#### Main prevalence study (RQ 3)

##### Non-responder analysis

For the responder analysis, the response rates were compared across the study centres and the four stratified age and gender groups. To assess the effect of the predictors, age, gender and centre on the response rate, a weighted logistic regression (responder/non-responder) was calculated, including the variable centre, gender and age and their interactions (including 3-way interactions). Thereby, weights were based on the number of contacted persons with regards to the response rate analysis. Backward elimination was carried out using likelihood ratio tests.

##### Analysis of sociodemographic characteristics

An analysis of the sociodemographic characteristics of the MentDis_ICF65+ sample included a descriptive analysis of the following variables: age, gender, place of birth, education, marital status, children and grandchildren, social relationships, employment status, socio-economic status, and religious affiliation for the total sample and the sample of each study centre separately. Univariate analyses of variance and chi-square tests were carried out to assess differences in the sociodemographic characteristics between the study centres.

##### Representativeness analysis

To assess the comparability of the recruited sample with the general community population from each study centre, the following descriptive comparisons were made: MentDis65+ sample vs. catchment area and vs. country population separately for each country (study centre), respectively. The comparison data were obtained from the following sources: Ferrara (Italy) — Italian National Institute of Statistics, 2011 Census (www.dati.istat.it); Geneva (Switzerland) — country data: Swiss Federal Statistical Office, Census 2000/2010 (www.bfs.admin.ch/bfs/portal/en/index.html), catchment area data: Cantonal Statistics Office of Geneva (www.ge.ch/statistique/welcome.asp); Hamburg (Germany) — Federal Statistical Office and Statistical Offices of the Länder, Census 2011 (http://www.statistik-portal.de/Statistik-Portal/GenesisUebersicht.asp); Jerusalem (Israel) — Central Bureau of Statistics Israel, Census 2011 (www.cbs.gov.il); London (England) — Office for National Statistics, Census 2011; Madrid (Spain) — Instituto Nacional de Estadistica, Population and Housing Census 2011 (www.ine.es). The following sociodemographic variables were compared: work status, marital status, number of children, education, number of household members, and place of birth. For some variables, no data were available for the catchment area, country population or both, and a representativeness analysis could not be completed. For Israel, only comparison data for the catchment area population was available (not for the country). No weighting procedures were applied. All analyses were computed using Predictive Analysis Software (PASW) version 18 [36].

##### Power calculation

To answer the main research question (RQ 3) on the prevalence of mental disorders in older adults within the cross-sectional study, an a priori power calculation was conducted. The required sample size was calculated using an expected prevalence rate of 30% based on reported lifetime prevalence rates of mental disorders from all age groups and countries. The expected standard error (SE) was set at 0.8%, and the expected width of the 95% confidence interval (CI) was set at ± 1.7%. Accordingly, an expected SE of 2.0% and an expected CI of ± 4.1% yield a sample size of *n* = 500 participants per country. Hence, an overall sample size of *n* = 3000 participants was needed. With a power of 80% or 90%, the minimum difference in the prevalence rates between two pre-specified countries that can be detected is 9.2% (from 34.6 to 25.4%, risk reduction 32.0%) or 9.4% (from 34.7 to 25.3%, risk reduction 36.2%), respectively [17].

##### Calculation of prevalence rates

To address RQ 3, survey analyses were conducted using post-stratification weights according to the number of inhabitants in each country and were stratified by gender and two age groups: 65–74 and 75–84-year olds. The adjusted lifetime, the 12-month and current prevalence rates and 95% confidence limits were estimated as the marginal mean from a weighted logistic regression adjusting for age in 5-year intervals, sex and study centre [37]. Group differences were tested using the main effect *p*-value of the model. Odds ratios (OR) and 95% confidence limits were also reported. All analyses were computed using Stata 12.1 [38].

## Results

### Feasibility of the CIDI65+ (RQ 1)

A sample of *n* = 18 participants aged 61 to 85 years with and without mental disorders in two study centres in Hamburg (Germany) and London (UK) were interviewed to assess the feasibility of the CIDI65+. Consequently, 179 problems were detected, 80% by one of the methods independently. The most frequent problems were usability problems (120), followed by acceptability (63) and programming (34) problems. Most of these problems were mild (146). Usability problems were for example missing words, typos or inconsistent format, acceptability problems included unclear or complicated questions, and programming problems were for example an inconsistency between the respondent booklet and the interview.

### Psychometric properties of the CIDI65+ (RQ 2)

A total sample of 228 participants, of which *n* = 68 participants completed both the test and retest interview, was analysed. The assessment of the test-retest reliability of the newly adapted CIDI65+ showed good results ranging between k = 0.55 for major depression and k = 1.00 for obsessive-compulsive disorder (k = 1.00). ICCs for the age of onset, recency, quantity, frequency and duration questions ranged between k = 0.60–0.90. Further details of the CIDI65+ psychometric properties are reported in Wittchen et al. [18].

### Main prevalence study (RQ 3)

#### Sampling

The sample was randomly selected from population registries (Italy and Germany) and from postal addresses of market research companies (England, Spain, Switzerland and Israel). The sampling frame and stages are presented in Table 1.

**Table 1:** Sampling frame and procedures in each country participating in the MentDis_ICF65+ study

	Hamburg (Germany)	Ferrara (Italy)	London (England)	Madrid (Spain)	Geneva (Switzerland)	Jerusalem (Israel)
Sample frame	Registries of resident registration offices in the metropolitan area of Hamburg through a market research company	Registries of resident registration offices in Ferrara and the province	Post office address files in Greenwich Borough and Canterbury District through a market research company	Addresses of residents in Madrid from the Statistical Institute of Madrid, a private company, and the Madrid City Council	Addresses of residents in the Geneva canton through a marketing company	Addresses of Jewish residents in the greater Jerusalem area through a market research company
Stage 1	Stratification by age and gender	Stratification by age and gender	Stratification by age and gender	Stratification by age and gender	Stratification by age, gender, socio-economic status, city/rural location	Stratification by age and gender
Stage 2	random selection from a registry of 5640 participants	random selection from registry - 1^st^ selection: 2000 participants - 2^nd^ selection: 1918 participants - 3^rd^ selection: 720 participants	random selection from files - 1^st^ selection: 2977 participants - 2^nd^ selection: 1145 participants	random selection from a census of 1072 participants (Madrid administration) and 1063 from files (private company)	random selection from registry - 1^st^ selection: 2000 participants - 2^nd^ selection: 700 participants	random selection - 1^st^ selection: 2500 participants - 2^nd^ selection: 708 participants

The total number of contacted individuals varied between 2534 older adults in London (England) and 5640 older adults in Hamburg (Germany). Participants from all study centres were approached with a written invitation letter and a phone call in all countries (except for Hamburg). In Hamburg, the participants had to respond in writing to participate. The contacted sample was called at least 5 times (10 times in Hamburg) at different times and days. In Geneva, if no phone number was available, a 2nd letter was sent. In Madrid, interviewers visited potential participants at home to ask for participation. Table 2 gives an overview of the contact procedure to the sample in each participating country, including rates and reasons for exclusion and drop out (Table 2). The total sample comprised *N* = 3142 older adult participants, who were interviewed face-to-face by trained lay interviewers with the CIDI65+ between January and October 2011.

**Table 2:** Contacting the sample in each country participating in the MentDis_ICF65+ study

	Hamburg (Germany)	Ferrara (Italy)	London (England)	Madrid (Spain)	Geneva (Switzerland)	Jerusalem (Israel)
Total number of contacted people (N)	5640	3213	2534	3375	2700	3208
Total number of people who agreed to participate (n, %)	626 (11.1)	546 (17.0)	542 (21.4)	584 (17.3)	565 (20.9)	609 (18.9)
Excluded participants (n)	13	23	10	3	4	60
Reasons for exclusion	cognitive problems	cognitive problems	cognitive problems	cognitive problems	cognitive problems	cognitive and language problems
Drop out (not required)^a^	106 (54)	0	36	26	41	7
Reasons for dropping out	Withdrawal of willingness to participate, illness, not reached, not required, invalid interview (incomplete data)	Denial or subsequent withdrawal of willingness to participate, illness, not reached, deceased, transferred, not required	Withdrawal of willingness to participate, illness, not reached, invalid interview (incomplete data)	Withdrawal of willingness to participate, not reached, illness, invalid interview (incomplete data)	Illness, holidays, difficulty locating, invalid interview (incomplete data)	Withdrawal of willingness to participate, illness
Incentives	25 Euro shopping voucher	A small gift	none	15 Euro shopping voucher	None, except for a choice of interview location (home/office)	a small gift
Final sample size (N)	510	524	496	555	520	542

^a^In Hamburg, n = 54 people had agreed to participate but were not contacted for an interview because the total sample size and the sample size in each stratum (age and gender groups) had been reached. They are listed as “not required”

#### Response rate

**Fig. 2:**
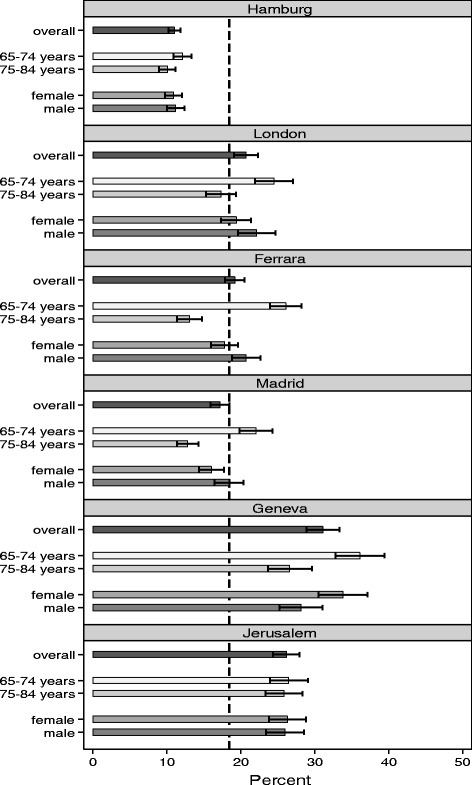
Results of the responder analysis for each study centre. *Detailed legend*: The response rate (in %) of the contacted sample was calculated by 2 age groups (65–74 years/ 75–84), gender (male/ female) and study centre (Hamburg/ Ferrara/ London/ Madrid/ Geneva/ Jerusalem). The dashed line shows the response rate for the total sample

The responder analysis shows significant differences in the response rate between the centres (*p* < 0.001). The lowest overall response rate was found for Hamburg with 11.1% (95%-CI [10.2; 11.9]). This also applies to all subgroups (regarding gender and age groups). The highest response rate was in Geneva with 31.0% (95%-CI [28.7; 33.2]). No gender effect was found (*p* = 0.738). However, there is a significant age effect, indicating that the response rate for younger participants is significantly higher than for older participants in all centres except Jerusalem (p < 0.001). In addition, a centre-specific effect was found for gender (*p* = 0.011) and both age groups (p < 0.001). The response rate of female and male participants differs significantly in Geneva (p = 0.011) and Ferrara (*p* = 0.031), whereby male participants responded more frequently in Ferrara and female participants in Geneva (see Fig. 2).

#### Interview location and duration

Eligible individuals were asked for their informed consent to participate in a face-to-face interview. Most interviews (81.6%) took place at the respondent’s home, 14.1% took place at the study centre premises, and a small number of interviews (4.2%) were conducted at other places (e.g., cafés).

The CIDI65+ assessment battery, including incorporated scales and mean administration times, are shown in Table 3. Overall, the interview lasted 95 min on average, with individual sections ranging between 2.7 (section F: bipolar disorders) and 17.4 (section A: sociodemography, screener) minutes.

**Table 3:** Socio-demographic characteristics of the MentDis_ICF65+ study by country

		Total sample n = 3142	Hamburg (Germany) *n* = 511	Ferrara (Italy) *n* = 518	London (England) *n* = 496	Madrid (Spain) *n* = 555	Geneva (Switzerland) *n* = 520	Jerusalem (Israel) *n* = 542	P-value
Age: mean (SD)		73.7 (5.6)	73.7 (5.0)	72.7 (5.3)	74.4 (5.6)	73.5 (6.0)	73.2 (5.6)	74.5 (5.6)	
Age categories: n (%)	65–74	1715 (54.6)	279 (54.6)	343 (66.2)	237 (47.8)	296 (53.3)	292 (56.2)	268(49.4)	
	>75	1427 (45.4)	232 (45.4)	175 (33.8)	259 (52.2)	259 (46.7)	228 (43.8)	274 (50.6)	
Gender: n (%)	Female	1592 (50.7)	249 (48.7)	233 (45.0)	275 (55.4)	288 (51.9)	265 (51.0)	282 (52.0)	
Born in the country where the interview took place categories: n (%)	Yes	2519 (80.2)	473 (92.6)	514 (99.2)	473 (95.4)	523 (94.2)	336 (64.6)	200 (36.9)	.001
Education: mean years of schooling (SD)		10.3 (3.2)	10.30 (2.0)	8.9 (2.0)	11.4 (1.7)	8.0 (4.1)	11.9 (1.9)	11.4 (2.7)	.001
Graduated from last school categories: n (%)	Yes	2392 (77.2)	485 (96.2)	439 (88.7)	342 (70.8)	297 (53.5)	433 (83.3)	396 (73.2)	.001
Marital status categories: n (%)	Married	1915 (61.0)	314 (61.7)	344 (66.5)	301 (60.7)	336 (60.5)	304 (58.5)	316 (58.3)	.003
	Separated, Divorced Widowed	1082 (34.5)	178 (35.0)	146 (28.2)	166 (33.5)	192 (34.6)	186 (36.8)	214 (39.5)	
	Never married/other	142 (4.5)	17 (3.3)	27 (5.2)	29 (5.8)	27 (4.9)	30 (5.8)	12 (2.2)	
Number of children: mean (SD)		2.3 (1.5)	1.9 (1.1)	1.7 (0.9)	2.3(1.4)	2.5 (1.4)	1.8 (1.2)	3.4 (2.0)	.001
Number of grandchildren: mean (SD)		3.9 (5.8)	2.4 (2.4)	1.6 (1.6)	3.8 (3.5)	3.0 (3.1)	2.6 (2.6)	9.6 (10.8)	.001
Number of household members: mean (SD)		0.8 (0.7)	0.7 (0.5)	1.0 (0.7)	0.8 (0.6)	1.0 (0.8)	0.8 (0.5)	0.8 (0.7)	.001
Number of close significants: mean (SD)		11.7 (11.2)	12.5 (10.9)	3.6 (4.2)	9.6 (9.2)	10.4 (9.2)	16.7(11.3)	17.4 (13.6)	.001
Employment status	Retired	2640 (84.6)	481 (94.9)	457 (90.5)	435 (88.4)	400 (72.2)	465 (89.4)	402 (74.4)	.001
	Working	227 (7.3)	14 (2.8)	15 (3.0)	45 (9.1)	13 (2.3)	50 (9.6)	90 (16.7)	
	Homemaker	224 (7.2)	10 (2.0)	27 (5.3)	5 (1.0)	137 (24.7)	5 (1.0)	40 (7.4)	
	Unemployed	8 (0.3)	0	1 (0.2)	0	4 (0.7)	0	3 (0.6)	
	Other	20 (0.6)	2 (0.4)	5 (1.0)	7 (1.4)	1 (0.2)	0	5 (0.9)	
Self-rated socioeconomic status: mean (SD)		6.2 (1.7)	6.6 (1.5)	5.8 (1.6)	6.4 (1.4)	5.5 (1.8)	6.9 (1.6)	6.1 (1.8)	
Self-rated financial situation categories: n (%)	Very good	356 (11.4)	66 (13.0)	11 (2.1)	68 (13.8)	15 (2.7)	122 (23.5)	71 (13.8)	.001
	Good	1372 (43.8)	241 (47.3)	148 (28.8)	254 (51.4)	150 (27.0)	335 (64.4)	244 (45.4)	
	Sufficient	1145 (36.6)	184 (36.1)	303 (58.9)	150 (30.4)	291 (52.4)	51 (9.8)	166 (30.9)	
	Poor	219 (7.0)	14 (2.8)	44 (8.6)	20 (4.0)	86 (15.5)	11 (2.1)	44 (8.2)	
	Very poor	37 (1.2)	4 (0.8)	8 (1.6)	2 (0.4)	13 (2.3)	1 (0.2)	9 (1.7)	
Self-rated religious affiliation categories: n (%)	Very important	827 (26.5)	106 (20.8)	199 (38.9)	116 (23.5)	186 (33.5)	75 (14.5)	145 (27.0)	.001
	Somewhat important	957 (30.6)	179 (35.2)	157 (30.7)	151 (30.6)	168 (30.3)	126 (24.3)	176 (32.7)	
	Not very important	660 (21.1)	117 (23.0)	85 (16.6)	105 (21.3)	127 (22.9)	144 (27.7)	82 (15.2)	
	Not at all important	682 (21.8)	107 (21.0)	70 (13.7)	122 (24.7)	74 (13.3)	174 (22.5)	135 (25.1)	

#### Socio-demographic characteristics of the MentDis_ICF65+ sample

Table 3 displays the socio-demographic characteristics of the MentDis_ICF65+ sample overall and by country. The mean age of the total sample was 73.7 years (SD = 5.6 years), and half of the sample was female (50.7%) as an effect of the stratification. The participants had attended school for a mean of 10.3 years (SD = 3.2 years). Most participants were married (61%), 35% were separated, divorced or widowed and 5% had never been married (Table 5). Approximately 85% of the participants were retired, with the lowest rates found in Spain (72%) and Israel (75%). About half of the participants rated their financial situation as good or very good (55%), with 8% rating it as poor or very poor. Compared to other study centres, in Madrid, the largest number of people rated their financial situation as poor (15.5%) or very poor (2.3%). About half of the participants rated their religious affiliation as somewhat or very important (57%). We found significant differences between the study centre samples with a few numerically apparent differences: In Jerusalem, the number of people born in the country where the interview took place was much lower (37%) than in other study centres (between 65 and 99%). Furthermore, differences appeared for the number of grandchildren (9.6 in Jerusalem, vs. 1.6–3.6 in all other study centres), number of close significants (3.6 in London vs. 16.7 and 17.4 in Geneva and Madrid), retired participants (72% in Madrid vs. up to 95% in Hamburg) and self-rated financial situation (“very good” 2.1 and 2.7% in London and Madrid vs. 23.5% in Geneva) (see Table 3).

#### Representativeness

The results of the comparison of the MentDis_ICF65+ sample with the catchment area and country populations of the study centres are displayed in Tables 4 and 5. The selection of the variables for this comparison was based on the sociodemographic characteristics described above, with the exception of the variables of number of grandchildren, financial status and religious affiliation, for which no comparison data were available. We found significant differences between the MentDis_ICF65+ sample and the catchment area (CA) population for the sociodemographic characteristics of work, marital status (except for Ferrara, *p* = .17; Jerusalem, *p* = .45), and Madrid, *p* = .37), number of children (no data available for Ferrara, Geneva, London and Madrid), education (except for Jerusalem and London; no data available), number of household members (except for Ferrara and Jerusalem; no data available), and having been born in the country where the interview took place (except for Ferrara; no data available).

**Table 4:** The results of the representativeness analysis for the MentDis65+ sample and the catchment area population for each study centre

		Hamburg (Germany)			Ferrara (Italy)			London (England)		Madrid (Spain)			Geneva (Switzerland)			Jerusalem (Israel)	
		Ment- Dis *n* > 489	Catchment *n* > 447,780		Ment-Dis *n* > 516	Catchment *n* > 77,598		Ment-Dis *n* > 448	Catch-ment *n* > 55,474	Ment-Dis *n* > 554	Catchment *n* > 412,849		Ment- Dis *n* > 453	Catchment *n* > 15,110		Ment-Dis *n* > 540	Catch-ment *n* > 50,698
Work status: retired (%)		94.5%	90.6%		88.4%	95.8%		88.4%	84.3%	72.1%	52.4%		89.4%	93.1%		74.4%	81.7%
Marital status: married		61.7%	58.1%		66.5%	64.%		60.8%	65.5%	60.5%	59.8%		58.5%	53.8%		58.5%	53.8%
Number of children	none	No data			No data			No data		No data			No data			5.0%	6.4%
	1															4.8%	6.6%
	2															20.3%	29.6%
	3															30.1%	20.7%
	4															19.7%	13.6%
	5															9.6%	9.2%
	6															3.3%	5.0%
Education: years of schooling		–			5	33.3%	70.6%	No data		7	40.5%	59.9%	7	3.5%	17.6%	No data	
		–			8	21.3%	16.7%			9	14.8%	0%	9	9.2%	19.1%		
		10	67.%	84.2%	10	5.6%	2.8%			–			–				
		13	32.%	15.8%	13	39.7%	9.9%			13	44.6%	40.1%	13	87.3%	63.2%		
Number of house-hold members	alone	32.7%	30.2%		No data			32.8%	79.5%	24.2%	21.6%		25.6%	50.6%		No data	
	2 people	65.3%	60.9%					67.2%	20.5%	54.2%	47.2%		68.4%	33.1%			
	3 people	2.0%	8.9%					–	–	18.1%	21.7%		6.0%	16.3%			
	4 people	–	–					–	–	3.2%	9.5%		–	–			
Born in the country where the interview took place		92.7%	85.5%		No data			95.4%	70.9%	95.9%	97.7%		68.5%	76.6%		36.9%	28.1%

**Table 5:** The results of the representativeness analysis for the MentDis65+ sample and the country population for each study centre

		Hamburg (Germany)			Ferrara (Italy)			London (England)		Madrid (Spain)			Geneva (Switzerland)			Jerusalem (Israel)	
		Ment-Dis sample *n* > 499	Country *n* > 12,221,270		Ment-Dis sample *n* > 508	Country *n* > 2,300,636		Ment-Dis sample n > 448	Country *n* > 637,979	Ment-Dis sample *n* > 513		Country *n* > 3,536,445	Ment-Dis sample n > 453	Country *n* > 1,258,927		Ment-Dis sample	Country
Work status: retired (%)		94.5%	91.9%		90.1%	88.4%		88.4%	86.0%	72.1%		57.9%	89.4%	93%		No data	
Marital status: married		61.7%	41.0%		66.5%	63.1%		60.8%	63.5%	60.5%		59.7%	58.5%	57.6%		No data	
Number of children		No data			No data			No data		No data			No data			No data	
Education: years of schooling					5	33.3%	70.6%	No data		7	40.5%	69.%	7	3.5%	15.2%	No data	
					8	21.3%	16.7%			9	14.8%	0%	9	9.2%	19.0%		
		10	67.3%	85.7%	10	5.6%	2.8%			–	–	–	–	–	–		
		13	32.7%	14.3%	13	39.7%	9.9%			13	44.6%	30.4%	13	87.3%	65.8%		
Number of household members	alone	32.7%	28.4%		23.8%	35.1%		32.8%	71.5%	24.4%		22.2%	25.6%	44.7%		No data	
	2 people	65.3%	61.6%		60.6%	46.6%		67.2%	28.5%	54.2%		47.3%	68.4%	39.3%			
	3 people	2.0%	10.0%		15.6%	18.3%		–	–	18.1%		20.9%	6.0%	16.0%			
	4 people	–	–		–	–		–	–	3.2%		9.5%	–	–			
Born in the country where the interview took place		92.7%	84.1%		99.4%	99.3%		95.4%	90.5%	95.5%		97.7%	68.5%	76.6%		No data	

With regard to the comparison of the MentDis_ICF65+ sample and the country population we found significant differences for three centres for the sociodemographic variables: work (except for Ferrara, *p* = .11 and London *p* = .06; Jerusalem, no data available), marital status (except for Ferrara, p = .06; Geneva, *p* = .36; London, *p* = .12; and Madrid, p = .36; Jerusalem, no data available), marital status (except for Ferrara, p = .06, d = .001; Geneva, p = .36, d = .001; London, p = .12, d = .001; and Madrid, p = .36, d = .001; Jerusalem, no data available), education (except for Jerusalem and London; no data available); number of household members (except for Jerusalem; no data available) and having been born in the country where the interview took place (except for Ferrara, *p* = .51; Jerusalem, no data available). Although most study centre samples differed from the population of their catchment area and/ or their country, most differences were numerically small (see Table 4; Table 5).

## Discussion

This paper describes the methodology of the MentDis_ICF65+ study, which is the first study to use an age-appropriate, standardized and structured clinical interview to assess the prevalence of a range of mental disorders according to the DSM-IV in older, community-dwelling adults in England, Germany, Israel, Italy, Spain and Switzerland. The theoretical framework of the ICF is used as a comprehensive understanding of the health status of older people. Prior to the cross-sectional survey to investigate the prevalence of mental disorders in older people, a pre-test and a pilot-test phase were conducted to ensure feasibility and psychometric soundness of the newly adopted interview.. In the pre-test phase the applied multi-method approach proved as an indispensable step, that identified problems with the interview’s acceptability, usability and programming and allowed to solve these problems prior to the field survey. The age-adapted CIDI65+ can be regarded as a feasible and reliable instrument for the assessment of most mental disorders in older adults [18]. Subsequently, in the cross-sectional survey, a homogenous sampling approach across study centres was implemented, and stratification allowed for a similar power across age and gender groups, which was particularly relevant for the smallest subsample group of 80- to 84-year-old men. However, stratification differed in Switzerland, where this was also done by socioeconomic status. We were able to implement a harmonized approach in contacting the sample and conducting the survey, i.e., contact by phone (except for Hamburg) and mail, standardized interviewer training, implementation of a standardized study protocol for all centres, and the use of stringent, high-quality data control procedures.

The response rate in our study varied from 31% in Switzerland to 11% in Germany. Significant gender (higher response rates from males in Spain, Italy and England; lower response rates from females in Switzerland) and age (higher for 65–74 age group in all centres) differences might result from diverse sample access across the centres. For example, the exclusively written contact regulations (without phone calls) in one study centre (Hamburg, Germany) due to ethical regulations may have led to an overall lower response rate compared to all other centres, which used a combination of letters and phone calls. The overall response rate of 20% is comparable to that of previous studies with similar recruitment procedures [39]. It may be possible that the low response rate was also associated with the fact that the study focused only on mental disorders. However, we tried to address the potential effect of negative attitudes towards mental health issues by labelling the study “well-being in older adults”. Moreover, it has consistently been noted in previous years that response rates in epidemiological surveys decline; however, nonresponse bias remains relatively small [39]. From a theoretical perspective, the bias of nonresponse could lead to an over- or under-estimation of the prevalence of mental disorders. Eaton et al. [40] and de Graaf et al. [41] reported that non-respondents have higher rates of mental disorders than respondents [41], while Alonso et al. [42] found higher prevalence rates of mental disorders in countries with lower participation rates. Keeter et al. [39] found few differences in estimates produced by a standard survey and by using more rigorous techniques aiming for a high rate of response.

One advantage of our study was that interviewers visited the respondents’ homes to conduct the interview; hence, a possible bias due to physical or mobility impairment may have been reduced. Due to the stratification, we achieved an almost equal distribution of age and gender groups across the whole sample. We found significant differences between the different study centre samples with regard for the place of birth, number of grandchildren, close significants, retirement and self-rated financial situation. A possible study centre effect needs to be remembered when interpreting our results. Although most study centre samples differed statistically from the population of their catchment area and their country, most differences were numerically small with small associations.

Some limitations need to be critically discussed with regards to the generalizability of our findings. Our results are limited by the exclusion of older people with cognitive impairments, those who are homeless and those who are unable to communicate in the languages used to conduct the study interviews. Additionally, we did not specifically intend to recruit older people living in care homes. The needs for representativeness have been critically discussed [43, 44] and the unknown added risk of bias of unmeasured variables due to the low response rate needs to be critically kept in mind, when interpreting the findings. Furthermore, the comparison data of the different catchment areas and countries varied due to a number of factors: availability of regional data (e.g., in Italy, data from north-eastern Italian region of Ferrara; in Israel, only Jewish inhabitants of Jerusalem), old age group-specific data (e.g., in Switzerland, the living situation of the general population excluding those aged 65 and above), and availability of the current data (e.g., in Switzerland, data from year 2001; in Spain, from 2001). There was also great variability in the sample size of the comparison data: The sample size of the comparison data was much larger than the MentDis_ICF65+ sample, leading to statistically significant differences, although numerically most of these differences were small.

## Conclusion

In summary, the methodology described above offers a novel approach: for the first time, an age-appropriate, reliable, structured and standardized instrument provides a diagnosis on mental disorders according to DSM-IV criteria of older adult participants in catchment areas of European and associated countries. This makes the MentDis_ICF65+ study a unique and important database on the prevalence of mental disorders and, moreover, offers insight into related factors including service utilization, quality of life, and impairment of activities and participation in older adults.

## Abbreviations

ANOVA: Analysis of Variance
BFI-10: Big Five Inventory
CA: Catchment Area
CAPI: Computer-Assisted Personal Interview
CI: Confidence Interval
CIDI65 +: Composite International Diagnostic Interview
DSM: Diagnostic and Statistical Manual of Mental Disorders
HoNOS65 +: Health of the Nation Outcome Scales65+
ICC: Intraclass Correlation Coefficient
ICD-10: International Classification of Diseases
ICF: International Classification of Functioning, Disability and Health
MMSE: Mini-Mental State Examination
NPV: Negative Predictive Value
OR: Odds Ratios
PASW: Predictive Analysis Software
PPV: Positive Predictive Value
RQ: Research Questions
SE: Standard Error
WHODAS II: World Health Organization Disability Assessment Schedule II
WHOQoL-BREF: World Health Organization Quality of Life Assessment- Abbreviated Version

## Footnotes

1 In the MentDis_ICF65+ study protocol [17], an erroneous cut-off score > 27 was reported. However, the cut-off score > 18 is correct.
